# The development of a gait shoe for multi-segment foot motion analysis

**DOI:** 10.1186/1757-1146-3-S1-O12

**Published:** 2010-12-20

**Authors:** Jill Halstead, Dennis McGonagle, Anne-Maree Keenan, Philip Conaghan, Anthony Redmond

**Affiliations:** 1 Section of Musculoskeletal Disease, Leeds Institute of Molecular Medicine, Leeds, UK; 2 Leeds NIHR Musculoskeletal Biomedical Research Unit, Leeds, UK

## 

Multi-segment kinematic foot models quantify foot motion in normal and pathological gait in mainly barefoot assessments, as shoes may confound the marker sets. The aim of this study was to provide a gait shoe to accommodate a multi-segment foot marker set without compromising shoe function. Fifteen normal volunteers and 15 patients with mechanical midfoot pain were recruited. All 30 participants undertook one gait analysis session in two conditions: barefoot and shod, in a random order. Markers were placed by a single clinician (JH) according to the Oxford multi-segment foot model, and kinematics were processed using Vicon Polygon. Foot kinematics differed between the normal and foot pain groups consistently in barefoot and shod conditions. At the hindfoot the foot pain group showed a similar pattern of eversion and decrease in dorsiflexion compared to normals. At the forefoot, the foot pain group showed greater dorsiflexion and less adduction. In the shod condition the differences between the participant groups were not as great. The results show the gait shoe has a minimal functional effect on the hindfoot as differences between groups remain. The gait shoe has some affect on the forefoot, but it did not change the kinematic pattern significantly compared to the normal group.

